# Body and wing size, but not wing shape, vary along a large-scale latitudinal gradient in a damselfly

**DOI:** 10.1038/s41598-021-97829-9

**Published:** 2021-09-20

**Authors:** David Outomuro, Maria J. Golab, Frank Johansson, Szymon Sniegula

**Affiliations:** 1 Department of Biological Sciences, University of Cincinnati, Rieveschl Hall, Cincinnati, OH 45221 USA; 2 Section for Animal Ecology, Department of Ecology and Genetics, Evolutionary Biology Centre, Uppsala University, Norbyvägen 18D, 75236 Uppsala, Sweden; 3 Institute of Nature Conservation, Polish Academy of Sciences, Kraków, Poland

**Keywords:** Biogeography, Ecology

## Abstract

Large-scale latitudinal studies that include both north and south edge populations and address sex differences are needed to understand how selection has shaped trait variation. We quantified the variation of flight-related morphological traits (body size, wing size, ratio between wing size and body size, and wing shape) along the whole latitudinal distribution of the damselfly *Lestes sponsa*, spanning over 2700 km. We tested predictions of geographic variation in the flight-related traits as a signature of: (1) stronger natural selection to improve dispersal in males and females at edge populations; (2) stronger sexual selection to improve reproduction (fecundity in females and sexual behaviors in males) at edge populations. We found that body size and wing size showed a U-shaped latitudinal pattern, while wing ratio showed the inverse shape. However, wing shape varied very little along the latitudinal gradient. We also detected sex-differences in the latitudinal patterns of variation. We discuss how latitudinal differences in natural and sexual selection regimes can lead to the observed quadratic patterns of variation in body and wing morphology via direct or indirect selection. We also discuss the lack of latitudinal variation in wing shape, possibly due to aerodynamic constraints.

## Introduction

The distribution ranges of species change over space and time and the processes that lead to such dynamic ranges have received major interest in biogeographical studies^1–3^. The study of the edges of distribution ranges is of particular interest, since it can inform on the resilience of species and their populations to environmental variation^4,5^. Edge populations experience the extremes of species ecological gradients, are usually smaller and more fragmented, and can be subject to different selection regimes than the core populations^4,6^. Increased dispersal ability and larger investment in reproduction at edge populations are predicted by theory and largely supported by empirical studies^7,8^. Higher dispersal ability at edge populations can occur as a result of local natural selection and spatial sorting of migrating phenotypes^8–11^. Spatial sorting is an accumulation of phenotypes that facilitate higher rates of dispersal at expanding range edges, combined with assortative mating of those phenotypes at the edge front^9^. Sexual selection is predicted to be stronger at northern edge populations since mating seasons are shorter and there are less favorable reproductive conditions^10,12–14^. The same prediction is expected for southern edge populations, where mating seasons are also shorter due to high temperatures that can lead to overheating^13^. However, dispersal and reproductive traits can be subject to conflicting selection pressures at edge populations^15^, possibly limiting the range expansion process^7^. Moreover, intralocus sexual conflict can vary along a species distribution range, potentially leading to smaller sex differences in traits under relaxed selection at edge populations^16,17^. Exploring variation of morphological traits across large latitudinal ranges and the signatures of selection and spatial dynamics at edge populations is important to understand the evolutionary and ecological fate of species facing environmental change^18,19^.

In flying species, flight is a fundamental trait that impacts many of the aspects related to natural and sexual selection of the species^20–23^. Flight-related traits (i.e., morphological and physiological traits that impact flight performance) are predicted to change following environmental gradients, and differences at edge populations related to increased sexual selection and dispersal ability have been previously reported^11,24–27^. However, studies incorporating the whole distribution range of the species and both the south and north margins are rare^28,29^. Damselflies and dragonflies are an interesting group to ask questions on flight morphology along latitudinal ranges because they are experiencing rapid changes in their distribution ranges^30,31^. Moreover, their flight-related traits are not only impacted by selection at the adult stage^32^, but also by selection at the larval stage^33,34^.

**Figure 1:**
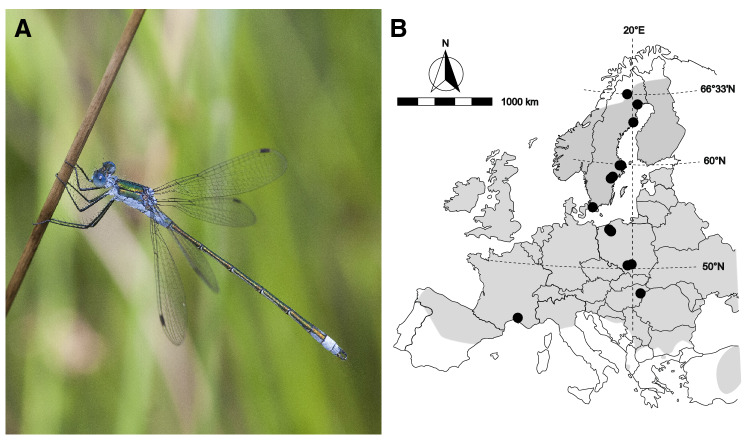
(**A**) Image of a male *Lestes sponsa* (credit: Bogusław Daraż). (**B**) Locations of the study populations (black dots) across the distribution range of *L. sponsa* (grey shade) in Europe (modified from Dijkstra and Schröter^35^). Map created using CorelDraw Graphics Suite 2021 (https://www.coreldraw.com/).

In this study, we used males and females of the damselfly *Lestes sponsa* (Hansemann, 1823) (Fig. 1A) to investigate the variation of morphological traits associated with flight across its entire latitudinal range. This damselfly is very well suited for investigating these questions. First, this species spans a large distribution range in Europe, from populations in northern Spain and Greece at the south margin, to populations in northern Sweden at the north margin^35^. Second, flight-related traits such as body size and wing morphology are subject to both natural (e.g., survival) and sexual selection (e.g., mating success) in this species^32^. Indeed, these selective pressures can reinforce or oppose each other when it comes to flight^32^: survival and mating success both favored intermediate body sizes; survival selection favored long and slender forewings and short and broad hindwings, whereas mating success favored short and broad forewings and narrow-based hindwings. Finally, *L. sponsa* is a strictly univoltine insect, i.e., one generation per year, that overwinters in the egg stage^36^. Thus, the fixed voltinism does not bias selection pressures on flight-related traits across latitudes^37^. At edge populations, individuals that disperse are predicted to be more abundant and hence dispersal-related flight traits should differ between core and edge population^26^. Males of *L. sponsa* show a mating system known as scramble competition, where males do not defend territories but actively search for females, can engage aggressively towards other males, and in the absence of available females will try to disrupt mating pairs^38^. Males of this species mate more intensively and invest more in armaments related to sexual selection (i.e., the male grasping apparatus that attaches to the female prothorax) at northern edge populations than in core or southern edge populations, presumably due to constraints imposed by short mating seasons^13,39^. Thus, we also expected stronger effects of sexual selection in traits related to flight in males at the northern edge populations compared the central core and southern edge populations^13^.

We studied four main flight-related morphological traits: body size, wing size, wing ratio (the proportion of wing size by body size) and wing shape. Regarding body size, previous studies in *Lestes* damselflies have shown that sexual selection (measured as mating sucess^32,38^) may favor intermediate male sizes, while larger sizes are generally expected to confer higher dispersal ability^40^. Under strong sexual selection, females would be predicted to show larger body sizes, since female body size is generally correlated with fecundity in insects^41^. Wing size is generally highly correlated with body size, but larger wing lengths and larger wing ratios can be related to dispersal ability^42–45^. Wing ratio also informs on relative investment on wing structures for a certain body size, which could be related to the aerodynamic needs of flight for each sex. Wing shape can also reflect both sexual selection pressures and dispersal ability. In males of *L. sponsa*, short and broad forewings (and to a lesser extent narrow-based hindwings), were favored by sexual selection, suggesting a role of flight maneuverability in mating success^32^. Moreover, wing shapes that are energetically more efficient can reflect dispersal ability, i.e., long and narrow wings^46,47^. In summary, we predicted latitudinal differences in these traits that would be the signature of both natural and sexual selection. More specifically, the flight-related traits should better reflect dispersal ability at the edge populations^26^, and therefore we predicted, for both sexes, larger body and wing size, larger wing ratio, and longer and narrower wing shapes at the northern and southern edges^40,42–47^. Alternatively, the signature of sexual selection could be stronger than dispersal selection for males at the northern and/or southern edge populations^13,39^. In that scenario, at edge populations males would show intermediate body sizes and short and broad forewings, favoring scrambling behavior^32,38^.

## Results

A total of 331 females and 337 males were collected across 17 populations spanning the entire distribution range of *L. sponsa* in Europe, from southern France to northern Sweden (Fig. 1B). The latitudinal variation of body size, wing size, wing ratio and wing shape were studied.

### Body size

**Figure 2:**
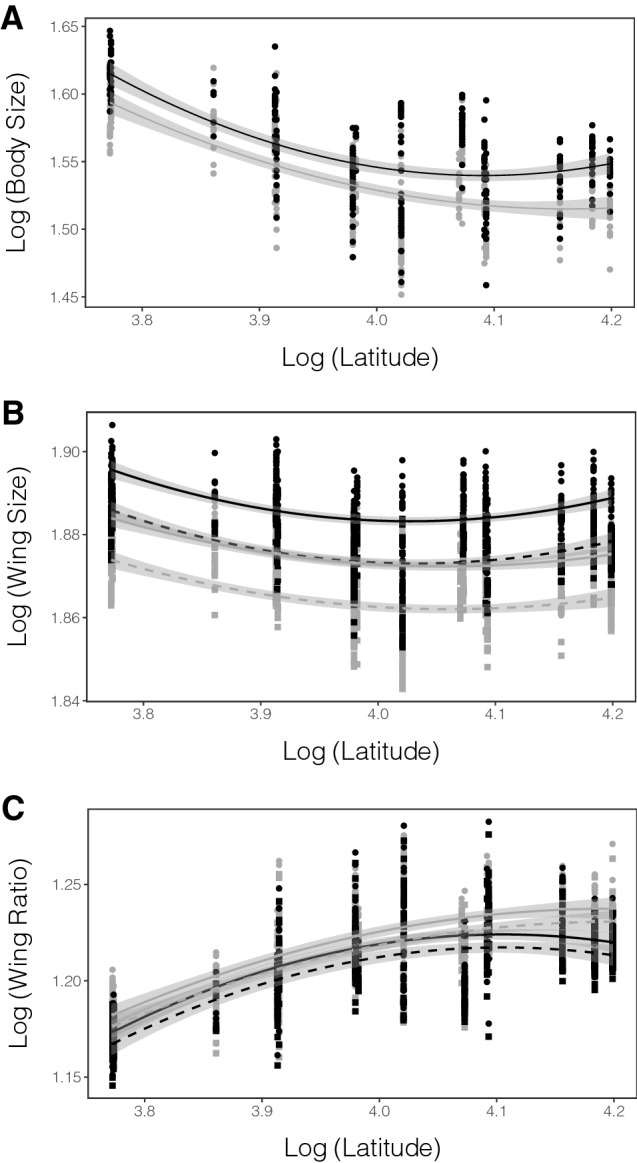
(**A**) Latitudinal variation of body size in *Lestes sponsa* (measured as head width, mm) for females (black) and males (gray). (**B**) Latitudinal variation of wing centroid size (derived from landmark coordinates in pixels) for females and males, including fore- and hindwings. (**C**) Latitudinal variation of wing ratio (ratio between wing centroid size and body size) for fore- and hindwings of females and males. (female forewings: black circles and solid line; female hindwings: black squares and dashed line; male forewings: gray circles and solid line; male hindwings: gray squares and dashed line; the shade around the regression lines shows the 95% CI).

The general linear model used to study the variation of body size (adjusted *R*^2^ = 0.47) showed that body size was larger in females than males (*F*_1,581_ = 80.09, partial *R*^2^ = 0.12, p < 0.001; mean ± SD, mm: females: 4.75 ± 0.17; males: 4.66 ± 0.17). Body size showed a slightly U-shaped pattern in relationship to latitude (linear term: *F*_1,581_ = 94.86, partial *R*^2^ = 0.14, p < 0.001; quadratic term: *F*_1,581_ = 88.64, partial *R*^2^ = 0.13, p < 0.001), with the highest values in the southernmost latitude, a steady decrease in the center, and a small increase again at the northernmost latitudes, especially for females (Fig. 2A). The relationship between body size and latitude did not differ between the sexes, since the interaction term sex × latitude was not significant (removed from the final model).

### Wing size

The effects of sex and latitude on fore- and hindwing size were first explored. The general linear models showed that wing size significantly differed between the sexes (Table 1A). Female wings were larger than male wings, and forewings were larger than hindwings in both sexes (mean ± SD, mm: female forewings: 156.77 ± 1.31; female hindwings: 155.18 ± 1.30; male forewings: 155.03 ± 1.28; male hindwings: 153.46 ± 1.28). Wing size also showed a significant U-shaped pattern in relation to latitude (Table 1A, Fig. 2B). Latitude explained 27.6% of the forewing size variation and 27.4% of the hindwing size variation (Table 1A). The shape of the quadratic curve of latitude slightly differed between the sexes (Fig. 2B), although this difference was not significant.

**Table 1:** A. Results of the General Linear Models inspecting the effects of latitude and sex on the variation of fore- and hindwing size in *Lestes sponsa*. B. Results of the General Linear Models inspecting the effects of latitude, body size (measured as head width) and sex on the variation of fore- and hindwing size in *Lestes sponsa*.

Effects	d.f.	SS	Partial *R*^2^	*F*	p (> *F*)
**(A) Not accounting for allometry**					
**Forewing size (full model *R***^**2**^** = 0.458)**					
Sex	1	0.900	0.368	383.930	< 0.001
Latitude	1	0.252	0.140	107.270	< 0.001
Latitude^2^	1	0.244	0.136	104.150	< 0.001
Residuals	659				
**Hindwing size (full model *R***^**2**^** = 0.467)**					
Sex	1	0.905	0.375	397.660	< 0.001
Latitude	1	0.243	0.139	106.920	< 0.001
Latitude^2^	1	0.236	0.135	103.520	< 0.001
Residuals	662				
**(B) Accounting for allometry**					
**Forewing size (full model *R***^**2**^** = 0.791)**					
Sex	1	0.271	0.334	291.317	< 0.001
Body size	1	0.861	0.614	923.978	< 0.001
Latitude	1	0.003	0.006	3.701	0.055
Latitude^2^	1	0.004	0.008	4.471	0.035
Residuals	580				
**Hindwing size (full model *R***^**2**^** = 0.789)**					
Sex	1	0.279	0.341	301.948	< 0.001
Body size	1	0.831	0.607	901.208	< 0.001
Latitude	1	0.005	0.008	4.932	0.027
Latitude^2^	1	0.005	0.010	5.717	0.017
Residuals	584				

The same general linear models were run accounting for the allometric component of wing size by incorporating body size into the models. Wing size showed a strong allometric component for both fore- and hindwings (Table 1B). The slopes of the regressions of wing size on body size were significantly larger than 1 both for fore- (slope ± 95% CI 1.36 ± 0.01) and hindwings (slope ± 95% CI 1.35 ± 0.09). When accounting for allometry, the effect sizes of latitude and its quadratic component accounted for less than 2% of the total wing size variation (Table 1B).

### Wing ratio

The two general linear models used to study the variation of fore- and hindwing ratio showed that this trait differed between the sexes (Table 2). Wing ratio was larger in males than in females for both wings (mean ± SD: male forewing: 1.22 ± 0.03; male hindwing: 1.21 ± 0.03; female forewing: 1.21 ± 0.03; female hindwing: 1.21 ± 0.03). Moreover, fore- and hindwing ratio showed a significant positive quadratic relationship with latitude (Table 2). Latitude and its quadratic component accounted for almost 20% of the total variation of wing ratio. Females showed lower values of wing ratio than males at the north edge populations (Fig. 2C), although this difference was not significantly different.

**Table 2:** Results of the General Linear Models inspecting the effects of latitude and sex on the variation of fore- and hindwing ratio in *Lestes sponsa*.

Effects	d.f.	SS	Partial *R*^2^	*F*	**p (> *F*)**
**Forewing ratio (full model *R***^**2**^** = 0.470)**					
Sex	1	0.010	0.006	3.319	0.069
Latitude	1	0.200	0.101	64.937	< 0.001
Latitude^2^	1	0.183	0.092	59.198	< 0.001
Residuals	581				
**Hindwing ratio (full model *R***^**2**^** = 0.458)**					
Sex	1	0.009	0.005	3.015	0.083
Latitude	1	0.185	0.093	60.108	< 0.001
Latitude^2^	1	0.169	0.085	54.692	< 0.001
Residuals	585				

### Wing shape

**Figure 3:**
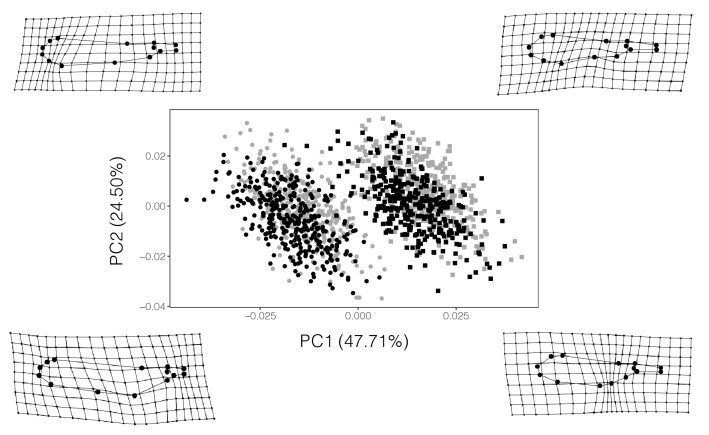
Wing shape variation of *Lestes sponsa* across all the populations studied. The thin-plate splines grids are exaggerated × 3 times for ease of visualization and show the extremes of variation for each corner of the plot. (female forewings: black circles; female hindwings: black squares; male forewings: gray circles; male hindwings: gray squares).

**Figure 4:**
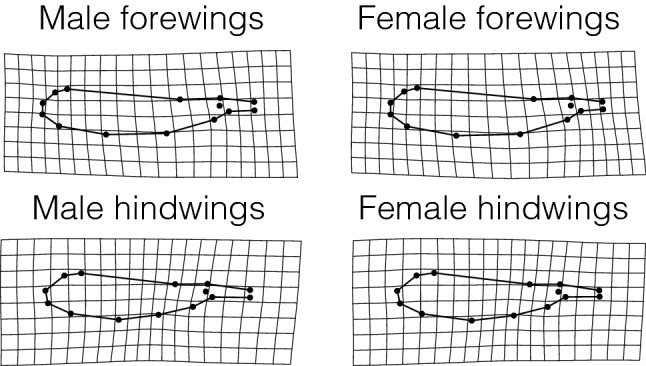
Thin-plate splines depicting the wing shape differences between males and females and fore- and hindwings of *Lestes sponsa*. The grids are exaggerated × 3 times for ease of visualization.

Wing shape was studied using geometric morphometrics (13 landmarks and 1 semi-landmark, see Supplementary Fig. S1). To visualize wing shape variation in the entire dataset of fore- and hindwings, a PCA on the wing shape variables was performed, and the variation of wing shape along the first two PCs (variance explained: PC1: 47.71%; PC2: 24.50%) was depicted as thin-plate spline deformation grids (Fig. 3). The plot of PC2 on PC1 showed large overlap between the sexes, but clear separation between fore- and hindwings (Fig. 3). Forewings were longer, slenderer and showed a shorter petiole; hindwings were shorter, broader and had a more elongated petiole (Figs. 3, 4).

Two Procrustes ANOVAs on the fore- and hindwing shape variables showed that the effects of sex and latitude accounted for less than 4% of the total wing shape variation (Table 3A). The differences in wing shape between the sexes were indeed subtle: female wings showed an overall slightly broader appearance and a slightly shorter petiole than male wings (Fig. 4, Supplementary Fig. S2). The deformation grids regarding wing shape variation along latitude are not shown since the wing shape variation was almost imperceptible.

**Table 3:** (A) Results for the Procrustes ANOVAs investigating the effects of latitude and sex on fore- and hindwing shape of *Lestes sponsa*. (B) Results for the Procrustes ANOVAs investigating the effects of latitude, size (measured as wing centroid size) and sex on fore- and hindwing shape of *Lestes sponsa*.

Effects	d.f.	SS	MS	*R*^*2*^	*F*	*Z*	p (> *F*)
**(A) Not accounting for allometry**							
**Forewing shape**							
Sex	1	0.009	0.009	0.036	25.605	4.951	< 0.001
Latitude	1	0.005	0.005	0.019	13.324	4.191	< 0.001
Latitude^2^	1	0.005	0.005	0.019	13.576	4.218	< 0.001
Residuals	659	0.234	0.0004	0.931			
**Hindwing shape**							
Sex	1	0.010	0.010	0.036	25.579	5.193	< 0.001
Latitude	1	0.005	0.005	0.019	13.382	4.163	< 0.001
Latitude^2^	1	0.005	0.005	0.019	13.695	4.192	< 0.001
Residuals	662	0.250	0.0004	0.930			
**(B) Accounting for allometry**							
**Forewing shape**							
Sex	1	0.004	0.004	0.016	11.538	3.917	< 0.001
Wing centroid size	1	0.006	0.006	0.023	16.850	4.489	< 0.001
Latitude	1	0.007	0.007	0.027	19.818	4.615	< 0.001
Latitude^2^	1	0.007	0.007	0.028	19.993	4.634	< 0.001
Residuals	658						
**Hindwing shape**							
Sex	1	0.004	0.004	0.016	11.923	4.075	< 0.001
Wing centroid size	1	0.004	0.004	0.016	11.904	4.040	< 0.001
Latitude	1	0.006	0.006	0.022	15.944	4.435	< 0.001
Latitude^2^	1	0.006	0.006	0.022	16.204	4.455	< 0.001
Residuals	661						

**Figure 5:**
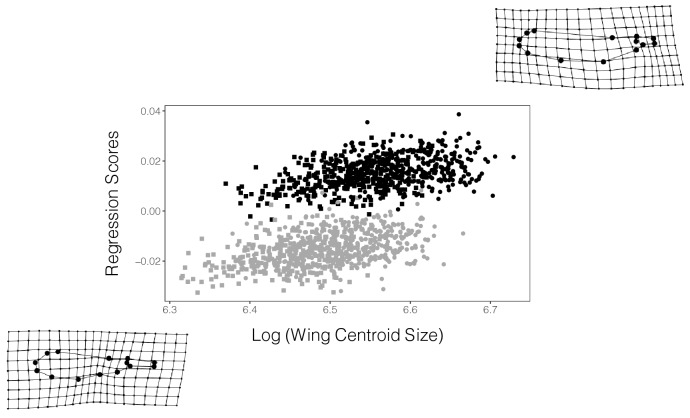
Allometric variation of wing shape of *Lestes sponsa*. Wing centroid size was derived from landmark coordinates in pixels. Fore- and hindwings of males and females are coded differently (female forewings: black circles; female hindwings: black squares; male forewings: gray circles; male hindwings: gray squares), but note that the regression slopes did not differ between these groups. Deformation grids depicting the extremes of allometric wing shape variation are included. The grids are exaggerated × 10 times for ease of visualization.

When the allometry of wing shape was accounted for by incorporating wing centroid size in the Procrustes ANOVAs (Table 3B), neither sex, latitude nor wing centroid size explained more than 5.5% of the total wing shape variation. Wing shape variation in relation to wing centroid size was subtle, with small wings showing a more pedunculated shape, broader tip, longer petiole and narrow wing base, and large wings showing a thicker shape with shorter petiole and broad base (Fig. 5).

## Discussion

We predicted that edge populations would differ in flight-related traits compared to core populations and that they should show trait values favoring dispersal ability and reproductive investment^7,8,13^. We found some support for these predictions. Our study highlights two main findings. We found that traits such as body size, wing size and wing ratio showed variation across the entire latitudinal range (contributing to 17–27% of the total variation of each trait), but wing shape showed very little variation across the same span (latitude contributed to less than 4% of the total variation of wing shape). We also observed differences between the sexes regarding the latitudinal variation of body size, wing size and wing ratio. We discuss below how the observed patterns for body and wing size agree with our predictions regarding selection favoring dispersal and fecundity in females, but not with selection on male sexual behaviors. We also discuss why wing shape might show very limited latitudinal variation.

Body size showed a U-shaped pattern in relationship with latitude. This relationship was especially clear for females, although the differences between the sexes were not statistically significant. In females, we predicted larger body sizes at the north and south edge populations to facilitate dispersal ability^40^ and increase their investment in reproduction via fecundity^41^. Our results agree with this prediction, but we also note that body size is much larger in the south than in the north edge populations. A plausible explanation is that body size in hemimetabolous insects such as damselflies is determined in part by the length of the season favorable for larval growth^28,37,48^: obligatory univoltine species such as *L. sponsa* usually tend to show a decrease in body size with latitude^37^, since the growth season decreases northwards. For males, we predicted that intermediate body sizes would be favored at edge populations (especially at the north edge^13^) due to their scrambling reproductive behavior^32,38^, but larger body sizes would be favored due to selection for dispersal ability. We found no support for this prediction since the results showed a similar body size at the north edge compared to the core populations, which could reflect conflicting selection pressures between sexual (scrambling behavior) and natural selection (dispersal). Similar to females, body size in males was the largest at the southern populations, probably due to the longer available growth season towards the south^37,49^. The observed differences in body size between the sexes along the latitudinal gradient, might be explained by latitudinal variation of sex-specific selection on body size. A recent study on the damselfly *Ischnura elegans* (Vander Linden, 1820) has found a decrease in sexual size dimorphism at north edge populations, as a result of an increase in male body size^16^. Our results show, however, a likely increase in female body size at north edge populations, resulting in an increase in sexual size dimorphism. One reason for these differences might be related to sex biases in dispersal: *Ischnura* damselflies can show male-biased dispersal^16^, while *Lestes* damselflies have been suggested to show female-biased dispersal^50^. Moreover, since females invest more in fecundity than males, females are probably under stronger fecundity selection at edge populations.

Our results for wing size in relation to latitude were similar to body size, a U-shaped pattern. Larger wing sizes can be related to better dispersal ability^42,43^, and our results suggest (similarly to our results for body size) that dispersal ability might be under stronger selection for females than males^50^, since the pattern was more accentuated in females. This latitudinal effect decreased from an effect size of 27% to less than 2% when allometric effects of body size were introduced in the model. Thus, our results suggest that selection on wing size might be acting through selection on body size.

Wing ratio showed a positive quadratic relationship with latitude, with significant differences between the sexes: wing ratio is lager in males than in females, and this difference is more pronounced at the north edge populations. Wing ratio has been also proposed as a proxy for better dispersal ability^43^, and we therefore predicted larger wing ratios at the north and south edge populations. However, our results showed a different pattern, with the smallest values of wing ratio at the south edge, an increase at core populations, and a different response between the sexes at the north edge populations. For females, the wing ratio values somewhat decreased compared to core populations, while for males, the values increased. Thus, wing ratio showed an inverse pattern to body size in relation to latitude. Part of the variation in wing ratio could be explained by the allometric relationship between wing size and body size. The allometric slopes between wing size and body size were larger than one for both fore- and hind wings, suggesting that either large-bodied damselflies invest more in larger wings or that smaller-bodied damselflies invest less in larger wings. It is likely that different latitudinal selection on body and wing size, and the particular aerodynamic needs of damselfly flight, are imposing the observed pattern in wing ratio.

Contrary to our expectations, wing shape showed very little variation in relation to latitude. We predicted a different wing shape at the edge populations compared to the core populations, favoring dispersal ability and reproduction^32,46,47^. However, latitudinal variation explained less than 5% of the total shape variation, regardless of considering or not the allometric component of wing shape. This is very interesting because flight traits such as wing shape are understood as the result of adaptive evolution driven by natural and sexual selection in many extant flying animal groups^20,51,52^. We suggest that the aerodynamic needs of each pair of wings for a given size of the individual might be under strong selection, probably differing between front- and hind wings^53–55^.

Our results showed that within the entire latitudinal range of a species, certain components of flight morphology, body size and wing size, might be under different selection regimes between core and edge populations, and that selection for dispersal and fecundity in females is probably stronger than sexual selection in males. The patterns observed at edge populations for body size and wing size might be a signature of a process of spatial sorting^9^. However, population genetics studies would be necessary to support this hypothesis.

## Methods

### Ethics declaration

The damselfly *L. sponsa* is considered a species of least concern by the IUCN red list of threatened species^56^. Damselfly collection in southern France was permitted by The Camargue Regional Nature Park, France. No other sampling permits were required for field collection.

### Field sampling and specimen preparation

Males and females of *L. sponsa* were sampled in 17 populations ranging from southern France to northern Sweden, covering the entire south-north distribution range of this species (Fig. 1B; see samples sizes in Table 4). Damselflies were collected during the peak of the flying season at each latitude (see sampling dates in Table 4). The altitude of the sampled populations ranged from 0 to 252 m a.s.l. (Table 4). These same damselflies were used in earlier cross-latitude comparison of adult size^37^. Animals were captured using a butterfly net and sacrificed in 70% ethanol. Head width was measured using a digital caliper to the nearest 0.01 mm. Head width is a commonplace measure of body size in damselflies and dragonflies, correlated with overall body size^57–59^. The measures of head width were log-transformed for use in further analyses. Fore- and hindwings were carefully removed and scanned in a flatbed scanner at 600 pixels/inch. The images of the wings were subsequently used for studying wing morphology. Individuals with torn or broken wings were discarded from the dataset.

**Table 4:** Sample size (N) of males and females of *Lestes sponsa* studied at each population including country of origin, sampling date, latitude and longitude, and altitude (head: head width, FW: forewings, HW: hindwings). The sample size for measurements of head width is variable depending on the corresponding measurements for fore- and hindwings of the same individual (N head FW-N head HW). No females were captured in the populations of Vistula Oxbow 1 Oświęcim (Poland) and Norrköping (Sweden).

Country	Population	Sampling date	Latitude	Longitude	Altitude (m a.s.l.)	Males			Females		
						N head	N FW	N HW	N head	N FW	N HW
France	Camargue nature reserve 1	30 June 2013	43.49216111	4.80943611	1	19–20	22	23	18	21	21
France	Camargue nature reserve 2	31 June 2013	43.53125278	4.77673611	0	19–20	22	23	20	24	24
Hungary	Halápi víztároló	4 July 2010	47.50638889	21.79694444	120	13	13	13	5	5	5
Poland	Vistula Oxbow 1, Oświęcim	23 July 2013	50.05341667	19.33433611	224	20	20	20	–	–	–
Poland	Vistula Oxbow 2, Oświęcim	21 July 2013	50.06162222	19.17104444	229	24	24	24	18	22	21
Poland	Extension of Pacwowy Pond II Błędów village	9 July 2014	50.12611111	19.17722222	252	18	18	20	19	19	19
Poland	Wetland close to Nadarzyce	28 July 2013	53.49394444	16.51554167	132	36–35	39	39	37–36	77	76
Poland	Tyczno wetland close to Komorze	26 July 2013	53.63838333	16.37433333	150	13	16	15	5	17	17
Sweden	Pond close to Lund 1	19–23 Aug 2013	55.73831667	13.15303056	22	14–15	14	16	11	11	11
Sweden	Pond close to Lund 2	19–23 Aug 2013	55.75146111	13.13564722	28	24	25	25	19	19	19
Sweden	Pond in Norrköping	3 Aug 2013	58.56505833	16.19680000	41	13	13	12	–	–	–
Sweden	Forest lake close to Åby	3 Aug 2013	58.71960000	16.29630556	124	22	22	22	23	23	23
Sweden	Pond in Uppsala	4 Aug 2013	59.84396389	17.66758056	4	18	18	18	16–17	16	17
Sweden	Pond in Bälinge	6 Aug 2013	59.935007	17.633479	13	21	21	21	20–21	20	21
Sweden	Nydalasjön lake, Umeå	6 Aug 2013	63.82490278	20.33363056	35	12	14	14	18	18	18
Sweden	Lake in Luleå	8–10 Aug 2013	65.60593333	22.12852500	7	11	11	13	22	22	22
Sweden	Pond in Jokkmokk	6 Aug 2013	66.60433889	19.88057500	220	20	20	19	17–15	17	15

### Wing morphology

To study wing size and shape, 13 landmarks and 1 semi-landmark along the wing outline and the arculus were digitized in tpsDig2^60^ (Supplementary Fig. S1). The landmark configurations were used to calculate the centroid size of each wing, i.e., the square root of the sum of squared distances between each landmark of the configuration and the centroid of the configuration. Centroid size was log-transformed for further analyses. Landmark configurations for all the wings were subject to Generalized Procrustes Analysis (GPA), in order to remove the effects of position, rotation and isometric size by minimizing the total sums-of-squared deviations of the landmark sets from all specimens to the average landmark configuration^61^. The semi-landmarks were permitted to slide along their tangent directions to minimize the Procrustes distance between each wing and the average configuration^62^. The GPA was first run for fore- and hindwings together (for visualization purposes), and then separately for fore- and hindwings. The aligned Procrustes coordinates resulting from the GPAs were projected into a linear tangent space to obtain the Procrustes shape variables. The sets of Procrustes shape variables for each separate wing (one for fore- and one for hindwings) were subsequently used as the variables representing wing shape variation. All the geometric morphometric analyses were performed in the R package geomorph v 3.3.2^63^.

### Variation of flight-related traits

Fore- and hindwing shapes can be subject to different flight constraints, so the statistical analyses were run separately for fore- and hindwings. The variation across latitudes of body size, wing size, wing ratio (the ratio between wing size and body size, calculated for fore- and hindwings) and wing shape was explored in separate models. Latitude was log-transformed before being entered in the models to improve normality and use similar scaling as for the other continuous variables. Altitudinal variation did not correlate with latitudinal variation (Pearson’s *r* = − 0.035). Altitude did not explain variation of body size, wing size or wing ratio (all cases, *R*^2^ < 0.003, p > 0.103), and was therefore excluded from the corresponding models. Altitude showed very little effects on wing shape variation (forewing shape: *R*^2^ = 0.017, p = 0.001; hindwing shape: *R*^2^ = 0.014, p = 0.001) so it was excluded from the final models.

For body size, fore- and hindwing size and fore- and hindwing ratio, separate general linear models were run, with sex as a fixed factor and latitude as a covariate. The interaction term was not significant and therefore removed from the final models. Visual inspection of the relationship between these variables and latitude suggested a possible quadratic relationship. Therefore, the models including the quadratic term of latitude were also run. The linear and the quadratic models were compared inspecting the ∆AIC using the R package MuMIn^64^. The models that included the quadratic term were selected over the models with only the linear term (body size: ∆AIC = 81.03; forewing size: ∆AIC = 95.25; hindwing size: ∆AIC = 94.73; forewing ratio: ∆AIC = 54.73; hindwing ratio: ∆AIC = 8.17). Finally, wing size can be affected by body size allometry. Therefore, another set of four general linear models including body size (measured as head width) as a covariate was also run. The non-significant interactions were removed one by one following a hierarchical approach, starting from the full model with all possible interactions, leading to no interactions present in the final model.

Wing shape variation was first visualized using Principal Component Analysis (PCA). The matrix of shape variables resulting from the full GPA (including fore- and hindwings together) was subject to a PCA, and the scores for PC1 and PC2 were used to plot wing shape variation. The variation of wing shape along each PC was depicted using thin-plate spline deformation grids. Wing shape variation for each wing was then analyzed using a Procrustes ANOVA on the shape variables resulting from the separate GPAs. These analyses were run in the R package geomorph^63^. The significance of the amount of shape variation attributable to each effect was evaluated using a residual randomization permutation procedure^65,66^. Latitude and sex and their interaction were included in the model. The interaction term was not significant and thus removed from the model. The fitting significantly improved by incorporating the quadratic term of latitude (p = 0.001), so this term was included in the final model. To account for the potential effects of allometry on wing shape, two more Procrustes ANOVAs were run incorporating also wing centroid size as a covariate.

## Supplementary Information

Supplementary Figures.

## Data Availability

The dataset including body size, wing size, wing ratio and wing shape variables is accessible from Zenodo Repository: https://zenodo.org/record/5500017#.YTtHbJNKjyg.
