# Supplementary material for: Body and wing size, but not wing shape, vary along a large-scale latitudinal gradient in a damselfly
Source: Sci Rep. 2021 Sep 20;11:18642. doi: 10.1038/s41598-021-97829-9 (PMC8452623; doi:10.1038/s41598-021-97829-9)
Supplement: Supplementary file 1 — Supplementary Figures. [file 41598_2021_97829_MOESM1_ESM.pdf]

SUPPLEMENTARY MATERIAL FOR

Body and wing size, but not wing shape, strongly vary along a large-scale latitudinal gradient in a damselfly

David Outomuro, Maria J. Golab, Frank Johansson and Szymon Sniegula

Corresponding authors:

David Outomuro

E-mail: outomuro.david@gmail.com

Szymon Sniegula

E-mail: sniegula@iop.krakow.pl

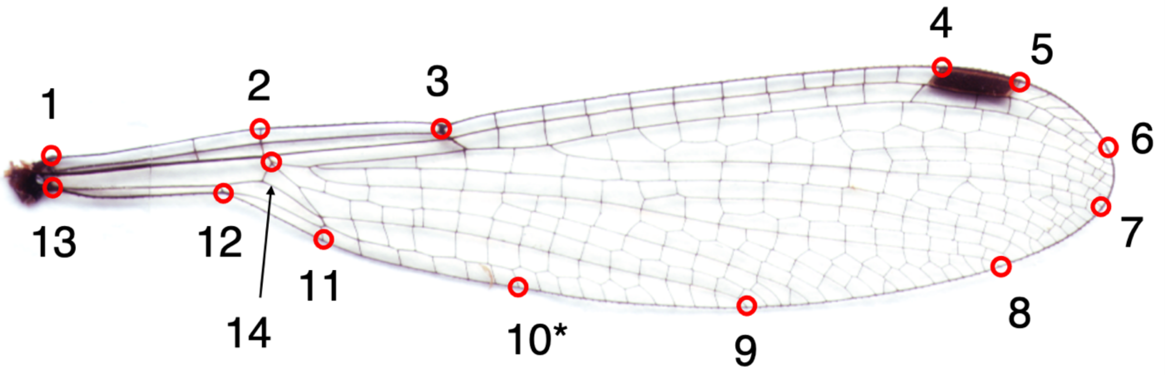

Fig. S1. Location of the 13 landmarks and the semi-landmark (*) used to study wing shape in *Lestes sponsa*.

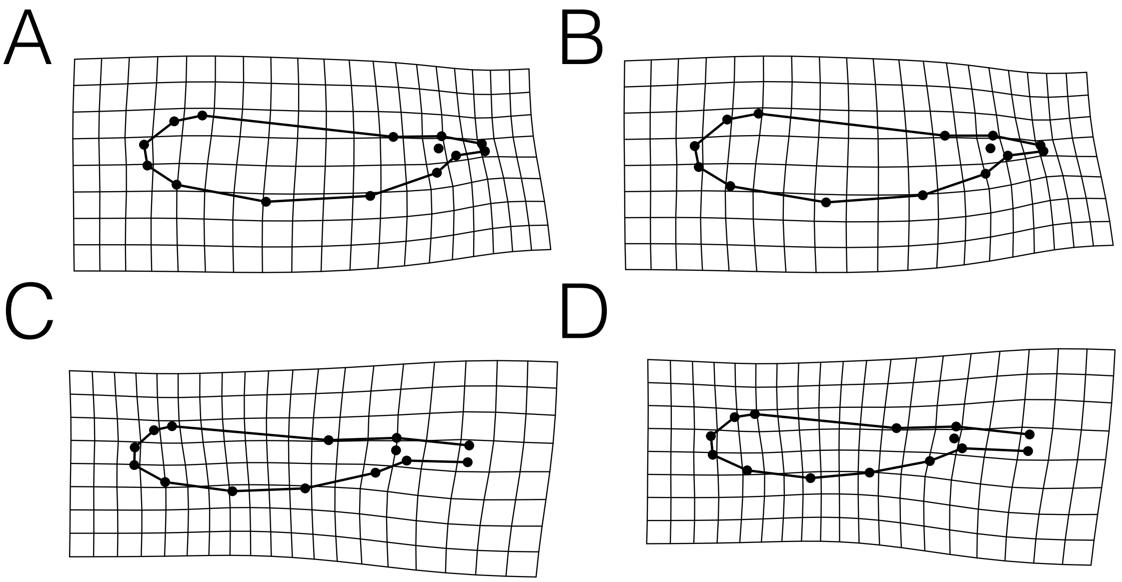

Fig. S2. Comparisons between male and female wings of *Lestes sponsa*. Mean shapes were compared to a selected reference mean shape. Female mean wing shape was first compared to male wing shape, and then male wing shape to female wing shape. A. Female forewing on male forewing. B. Female hindwings on male hindwing. C. Male forewing on female forewing. D. Male hindwing on female hindwing. The thin-plate splines are exaggerated x10 times for ease of visualization.
